# Functional Food Ingredients Enhancing Immune Health

**DOI:** 10.3390/ijms26178408

**Published:** 2025-08-29

**Authors:** Irene Skenderidou, Stefanos Leontopoulos, Prodromos Skenderidis

**Affiliations:** 1 School of MΕd, University of Nicosia, 46 Makedonitissas Avenue, CY-2417 Nicosia, Cyprus; skenderidou.e@live.unic.ac.cy; 2 School of Applied Arts and Sustainable Design, Hellenic Open University, Parodos Aristotelous 18, 26335 Patras, Greece; sleontopoulos@uth.gr; 3 School of Sciences & Technology (SST), Hellenic Open University, Parodos Aristotelous 18, 26335 Patras, Greece

**Keywords:** functional foods, immune system, immunomodulation, bioactive compounds, phytochemicals, micronutrients, probiotics

## Abstract

Functional foods enriched with bioactive compounds—including vitamins, minerals, polyphenols, probiotics, fatty acids, and amino acids—have gained growing attention due to their ability to modulate immune responses. This review aims to summarize and critically evaluate evidence from both preclinical and clinical studies on the immunomodulatory effects of these compounds. A structured literature search was performed across major scientific databases in accordance with PRISMA 2020 guidelines. Seventy studies met the predefined eligibility criteria and were included. Evidence indicates that functional ingredients support immune function via antioxidant, anti-inflammatory, and microbiome-mediated pathways. Clinical trials further report benefits including a reduced risk of respiratory infections and enhanced vaccine responses. Nonetheless, important challenges remain regarding bioavailability, inter-individual variability, and the long-term safety of supplementation. Emerging research on precision nutrition and innovative delivery systems may further enhance the efficacy of these bioactive compounds. Overall, functional foods and nutraceuticals show strong potential as adjunct strategies for maintaining immune health; however, further well-designed clinical studies are required to confirm their translational applicability.

## 1. Introduction

Over the past decade, the intricate relationship between dietary intake and immune function has attracted increasing scientific interest. This interest has been particularly driven by the expanding field of functional foods—dietary components that exert physiological effects beyond basic nutrition, especially in modulating immune responses and systemic inflammation [1,2]. A growing body of clinical and mechanistic evidence has highlighted the role of bioactive food constituents—including micronutrients, polyphenols, omega-3 fatty acids, probiotics, prebiotics, and specific dietary peptides—in enhancing immune surveillance, supporting mucosal defenses, and reducing immunopathology across diverse populations [3,4,5].

The emergence of the COVID-19 pandemic further emphasized the importance of nutritional status in immunological health. An increase in immunonutrition research demonstrated that dietary composition could influence susceptibility to infection, immune cell functionality, and vaccine responsiveness [6,7]. Key micronutrients such as vitamin D, vitamin C, zinc, and selenium are now recognized as pivotal cofactors in the regulation of both innate and adaptive immunity. Their biological functions include support of epithelial barrier integrity, modulation of cytokines, antioxidant defense, and enzymatic signaling [8,9]. Meta-analyses have shown that correcting micronutrient deficiencies—particularly through supplementation—may reduce the frequency and severity of respiratory infections [10].

In parallel, polyphenols—a structurally diverse class of plant-derived compounds encompassing flavonoids, stilbenes, and phenolic acids—are known to exert immunomodulatory and antioxidant effects. These phytochemicals modulate cellular signaling through pathways such as nuclear factor-kappa B (NF-κB), mitogen-activated protein kinases (MAPK), and nuclear factor erythroid 2 related factor 2 (Nrf2), affecting cytokine expression, oxidative stress, and immune cell differentiation [11,12]. Both epidemiological analyses and controlled intervention trials have linked consumption of polyphenol-rich foods (e.g., berries, tea, olive oil) to attenuated inflammatory responses and enhanced immune biomarkers, with consistent findings reported across preclinical animal models and human cohort studies [13].

The gut–immune axis has emerged as a pivotal interface for dietary–immune interaction. Probiotics—primarily from genera Lactobacillus and Bifidobacterium—and prebiotics such as inulin and fructooligosaccharides (FOS), have demonstrated significant immunomodulatory potential. These compounds alter gut microbiota composition, stimulate short-chain fatty acid (SCFA) production, and promote regulatory T cell (Treg) activation, thus enhancing mucosal immunity and promoting immune tolerance [14]. Recent studies also support the use of symbiotic (combinations of probiotics and prebiotics) in reducing infection risk and ameliorating inflammatory conditions [15].

Beyond microbial modulation, omega-3 fatty acids—notably eicosapentaenoic acid (EPA) and docosahexaenoic acid (DHA), abundant in marine sources—have demonstrated anti-inflammatory and pro-resolving properties. These fatty acids serve as precursors to specialized pro-resolving mediators (SPMs) that influence leukocyte recruitment, cytokine resolution, and macrophage polarization [16]. Clinical trials suggest that omega-3 supplementation may attenuate systemic inflammation and enhance immune homeostasis in older adults and individuals with chronic comorbidities [17].

Despite this growing promise, several challenges remain in the clinical translation of functional food strategies. These include inter-individual variability in bioavailability, influence of genetic and microbiota-related factors, lack of standardization in study design, and regulatory ambiguity regarding health claims [18]. Moreover, pronounced heterogeneity in intervention efficacy across demographic strata underscores the necessity for precision nutrition paradigms, informed by multi-omics biomarker profiling to capture individual immunophenotypes, comprehensive nutrient status indices, and integrative risk models for disease susceptibility.

This review aims to synthesize and critically evaluate the scientific literature from the past decade (2015–2025) concerning the use of functional food ingredients to support and enhance immune function. We explore mechanistic pathways, summarize clinical and preclinical data, and assess current applications and future directions in the field of immunonutrition and functional food development. This review differs from previous ones by providing an integrative overview of both traditional micronutrients and emerging bioactive compounds with immunomodulatory potential, while placing particular emphasis on clinical trial outcomes and translational considerations. In addition, we highlight knowledge gaps and opportunities for precision nutrition strategies, thereby offering a forward-looking perspective not typically covered in earlier reviews

## 2. Historical Evolution of Functional Food Ingredients in Immune Modulation (1925–2025)

The concept of modulating health through diet has been investigated deep in time, dating back to ancient medical traditions that emphasized the medicinal value of food. However, the scientific exploration of functional food ingredients and their role in immune health began to take shape in the early 20th century. Between the 1920s and 1950s, landmark research on vitamin deficiencies—particularly vitamins A, C, and D—uncovered their essential roles in immune development, epithelial barrier maintenance, and infection resistance. These discoveries laid the foundation for the field of nutritional immunology [19,20,21].

From the 1960s to the 1980s, research transitioned toward understanding the importance of trace elements and lipid mediators. Zinc, selenium, and iron were identified as critical cofactors in immune enzymatic reactions, lymphocyte proliferation, and redox balance. At the same time, dietary lipids, including omega-3 fatty acids, began to be investigated for their anti-inflammatory effects and their influence on prostaglandin synthesis [22,23,24].

During the 1990s and early 2000s, interest grew in polyphenols and phytochemicals due to their antioxidant capacity and ability to modulate key signaling pathways. Advances in molecular biology revealed that polyphenols could influence immune cell behavior via transcriptional regulation of NF-κB, MAPK, and Nrf2, linking dietary antioxidants to inflammation resolution and immune tolerance [25,26,27]. In parallel, the discovery of the gut microbiota as a critical regulator of immunity spurred interest in probiotics and prebiotics as modulators of mucosal and systemic immune responses.

Between 2010 and 2020, high-throughput technologies such as metabolomics, transcriptomics, and microbiome sequencing provided a more precise understanding of how bioactive food ingredients interact with host immune pathways. The concept of the gut–immune axis became central in immunonutrition, with synbiotics and dietary fibers studied for their ability to influence T-regulatory cells, dendritic cell maturation, and SCFA production [28,29].

The COVID-19 pandemic, beginning in 2020, catalyzed unprecedented research into the role of diet quality and nutritional status in modulating infectious disease susceptibility and immune resilience. Studies investigated both prophylactic and therapeutic contexts, highlighting the potential protective effects of bioactive compounds and nutrients—including vitamin D, zinc, quercetin, probiotics, and omega-3 polyunsaturated fatty acids—through mechanisms such as immunomodulation, attenuation of inflammatory responses, and enhancement of antiviral defense pathways [30,31,32]. This period also marked the integration of personalized nutrition and precision immunology, aiming to match specific dietary components with individual immunophenotypes [33,34].

In parallel, multi-omics approaches—integrating metabolomics, transcriptomics, proteomics, and metagenomics—have provided a comprehensive understanding of host–microbiota–immune interactions and the metabolic fate of functional compounds [35]. These tools enable precision nutrition strategies, tailoring interventions based on genetic polymorphisms, microbiota composition, epigenetic modifications, and inflammatory markers. Furthermore, emphasis is now placed on formulation optimization, enhancement of bioavailability (e.g., liposomal or nanoencapsulation systems), and regulatory harmonization to ensure efficacy and safety validation of functional food health claims [36,37].

## 3. Bioactive Compounds in Functional Foods and Their Impact on Immunity

**Figure 1:**
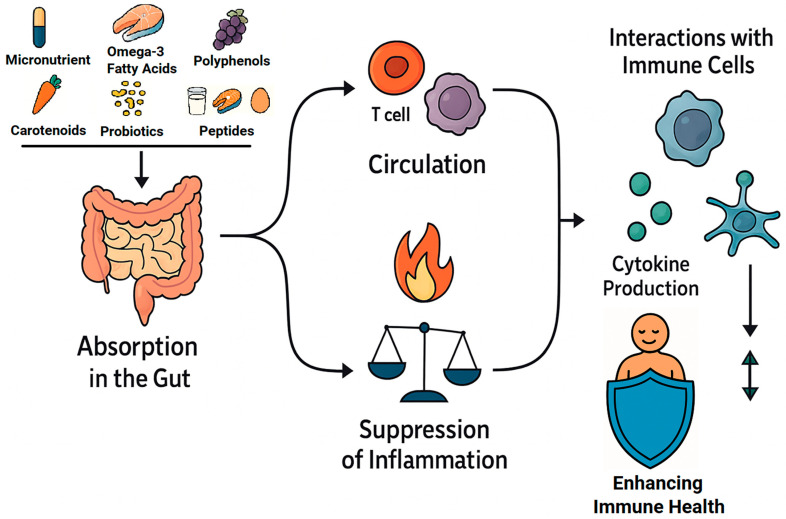
Mechanisms of action of functional ingredients in the immune system.

The intricate interplay between functional food bioactives and immune function is supported by growing mechanistic and clinical evidence—especially in the context of aging, inflammaging, and immune resilience. Functional compounds such as vitamins C, D, E, zinc, selenium, polyphenols, omega-3 fatty acids, and probiotics/prebiotics emerge as central modulators of both innate and adaptive immunity (Figure 1).

Their diverse chemical structures allow them to interact with multiple cellular targets and signaling pathways, as presented in Table 1, conferring a broad spectrum of immunological benefits that contribute to maintaining homeostasis and enhancing host defense mechanisms [38].

### 3.1. Micronutrients in Immune Modulation

Micronutrients, comprising essential vitamins and trace elements, serve as crucial cofactors in numerous enzymatic and cellular processes that underpin immune function. Adequate levels ensure the optimal activity of both innate and adaptive immune responses, which together maintain host defense and immunological homeostasis [39]. Deficiencies or imbalances in these micronutrients are associated with increased susceptibility to infections, dysregulated inflammatory responses, and impaired vaccine efficacy [2,40].

Vitamin D has garnered significant attention for its multifaceted immunomodulatory effects. Upon activation to its hormonal form, calcitriol (1,25-dihydroxyvitamin D3), it binds to the vitamin D receptor (VDR) expressed on various immune cells, including macrophages, dendritic cells, and T lymphocytes [41]. This interaction downregulates the production of pro-inflammatory cytokines such as interleukin (IL)-6, tumor necrosis factor-alpha (TNF-α), and interferon gamma (IFN-γ), while enhancing anti-inflammatory cytokines like IL-10 [42]. Vitamin D also promotes the differentiation and function of regulatory T cells (Tregs), facilitating immune tolerance and preventing excessive immune activation. Epidemiological and interventional studies have linked low vitamin D status to increased incidence and severity of respiratory infections, including SARS-CoV-2, underscoring its therapeutic potential [43,44,45].

Vitamin C (ascorbic acid) is a water-soluble antioxidant critical for maintaining redox balance within immune cells. It directly scavenges reactive oxygen species (ROS), protecting leukocytes from oxidative damage during respiratory burst activities [46]. In addition, vitamin C supports chemotaxis, phagocytosis, and microbial killing by neutrophils, and enhances lymphocyte proliferation and differentiation [47]. Clinical evidence indicates that vitamin C supplementation can reduce the duration and severity of upper respiratory tract infections, owing to its immune-potentiating properties [48]. Vitamin C also influences the gene expression of immune-related factors, including NF-κB, thereby modulating inflammatory pathways [49].

Vitamin E, a lipid-soluble antioxidant, protects polyunsaturated fatty acids in cell membranes from peroxidation, thereby maintaining the integrity and functionality of immune cells [50]. It enhances T cell-mediated immune responses and antibody production, with deficiency linked to increased susceptibility to viral infections [51]. Supplementation studies in elderly populations have demonstrated improvements in immune function and reduced incidence of infections upon vitamin E administration [52].

Zinc plays an indispensable role in immune competence through its involvement in DNA synthesis, cell division, and apoptosis of immune cells [53,54,55]. It is integral to thymic hormone activity and T cell maturation, influencing the balance between T Helper Cells Type 1 (Th1) and type 2 (Th2) responses [56]. Zinc deficiency results in thymic atrophy, lymphopenia, and impaired macrophage and natural killer (NK) cell functions [57]. Clinical trials have confirmed that zinc supplementation reduces the incidence, severity, and duration of infectious diseases such as pneumonia and diarrhea, particularly in pediatric populations [58].

Selenium is essential for the activity of selenoproteins, including glutathione peroxidases and thioredoxin reductases, which protect immune cells from oxidative stress and regulate redox-sensitive signaling pathways [59]. Selenium deficiency compromises the proliferative capacity of T cells and diminishes NK cell cytotoxicity, contributing to heightened viral pathogenicity [60]. Supplementation has been linked to enhanced immune surveillance and attenuation of excessive inflammation, making it a key micronutrient in functional foods aimed at immune support [61,62].

Iron is fundamental for immune cell metabolism and proliferation, serving as a cofactor in enzymes involved in DNA synthesis and energy production [63]. Both iron deficiency and overload adversely affect immunity; deficiency impairs lymphocyte proliferation and phagocytic activity, while excess iron can exacerbate oxidative stress and promote pathogen growth [64]. Functional foods enriched with bioavailable forms of iron are being developed to mitigate anemia-related immune dysfunction, especially in vulnerable groups [65].

### 3.2. Polyphenols and Flavonoids: Molecular Mechanisms and Immune Modulation

Polyphenols are a large group of secondary plant metabolites characterized by the presence of multiple phenolic rings. Their immunomodulatory effects are primarily attributed to their antioxidant properties, which involve scavenging free radicals and chelating metal ions, thereby preventing oxidative damage to immune cells and preserving the function of redox-sensitive transcription factors such as Nrf2 [66]. Activation of Nrf2 triggers the expression of cytoprotective genes encoding antioxidant enzymes like heme oxygenase-1 (HO-1) and glutathione peroxidase (GPx), which together reduce cellular oxidative stress and inflammation [67]. Beyond its role in immune modulation, Nrf2 has been recognized as a master regulator of cellular defense mechanisms, and its dysregulation has been implicated in the onset and progression of both cancerous and non-cancerous diseases [68,69]. This underscores the broader relevance of targeting Nrf2 pathways not only for immune-related outcomes but also for chronic disease prevention and therapy.

Polyphenolic compounds, including quercetin, epigallocatechin-3-gallate (EGCG), resveratrol, and curcuminoids, exhibit multifaceted immunoregulatory actions by modulating key age-related intracellular pathways such as AMP-activated protein kinase (AMPK), Sirtuin 1 (SIRT1), mechanistic target of rapamycin (mTOR), and NF-κB. These bioactives suppress the senescence-associated secretory phenotype (SASP), reduce oxidative stress, and preserve mitochondrial integrity [70,71]. Beyond redox regulation, polyphenols influence DNA methylation and histone modification, restoring youthful gene expression profiles in aging immune cells and reducing chronic inflammatory tone [72]. Animal and human studies demonstrate improved Treg cell balance, suppressed pro-inflammatory cytokines, and enhanced phagocytic activity following polyphenol supplementation [73,74,75].

EGCG, a well-studied polyphenol, exerts immunoregulatory effects by inhibiting the nuclear translocation of NF-κB, a master regulator of inflammation. This inhibition decreases the transcription of pro-inflammatory cytokines such as IL-1β, IL-6, and TNF-α, which are pivotal in chronic inflammatory and infectious diseases [76]. EGCG also modulates adaptive immunity by enhancing the differentiation of regulatory T cells (Tregs), which suppress excessive immune activation and promote tolerance, while concurrently reducing the differentiation of pro-inflammatory Th17 cells [77,78].

Curcumin, a principal curcuminoid in turmeric, exhibits broad immunomodulatory actions through inhibition of cyclooxygenase-2 (COX-2), inducible nitric oxide synthase (iNOS), and modulation of the Janus kinase/signal transducer and activator of transcription (JAK/STAT) and NOD-, LRR-, and pyrin domain-containing protein 3 (NLRP3) inflammasome pathways [79]. These effects lead to decreased production of inflammatory mediators and suppression of macrophage activation. Importantly, curcumin enhances the phagocytic capacity and antigen-presenting functions of macrophages and dendritic cells, which are essential for initiating effective immune responses [80]. Its ability to potentiate NK cell cytotoxicity further contributes to enhanced innate immunity against viral infections and tumor surveillance [81]. Curcumin also has shown promising therapeutic potential in the prevention and management of chronic diseases, including diabetes and certain types of cancer due to their potent antioxidant properties [82,83]. These findings suggest that curcumin may serve not only as a dietary component but also as an adjunct in disease prevention and therapy, although further well-designed clinical studies are needed to confirm its efficacy and safety.

Flavonoids such as quercetin and hesperidin display potent immunomodulatory and antiviral properties. Quercetin inhibits viral entry and replication by targeting viral proteases and blocking membrane fusion, while reducing mast cell degranulation and histamine release, thereby mitigating allergic inflammation [84]. It also modulates key signaling cascades, including MAPK and NF-κB, to decrease cytokine storms characteristic of severe viral infections [85]. Hesperidin, abundant in citrus fruits, promotes endothelial function and reduces vascular inflammation by enhancing nitric oxide synthesis and suppressing adhesion molecules such as ICAM-1 and VCAM-1, limiting leukocyte infiltration into inflamed tissues [86]. These effects are essential for controlling systemic inflammation and preventing immune-mediated tissue damage.

### 3.3. Carotenoids: Antioxidant Defense and Immune Enhancement

Carotenoids, a class of fat-soluble pigments, contribute to immune health through their antioxidant capacity, protecting immune cell membranes from lipid peroxidation and preserving membrane fluidity essential for receptor-mediated signaling [87]. Beta-carotene serves as a provitamin A, converting to retinol and retinoic acid, which regulate gene expression via retinoic acid receptors (RARs). These processes influence the development, differentiation, and function of T and B lymphocytes [88]. Retinoic acid also promotes the generation of gut-homing T cells and supports mucosal immunity, which is vital for the first-line defense against pathogens [89].

Lycopene and lutein demonstrate strong singlet oxygen quenching and ROS scavenging activity, reducing oxidative stress and modulating the expression of inflammatory cytokines such as IL-8 and monocyte chemoattractant protein-1 (MCP-1) in monocytes and macrophages [90]. Observational studies have associated higher plasma carotenoid levels with reduced systemic inflammation markers (e.g., C-reactive protein) and enhanced lymphocyte proliferation in elderly populations, suggesting a protective role in immunosenescence [91].

Furthermore, carotenoids modulate cellular signaling pathways, including NF-κB and MAPKs, which influence cytokine and adhesion molecule expression. These actions further contribute to immune regulation and the maintenance of immune homeostasis [92].

### 3.4. Omega-3 Fatty Acids and Immune Resolution

The biological functions of long-chain omega-3 polyunsaturated fatty acids (LC-PUFAs), particularly eicosapentaenoic acid (EPA) and docosahexaenoic acid (DHA), extend beyond structural membrane components, emerging as critical regulators of immune responses and inflammation resolution. These fatty acids are incorporated into cellular membranes, where they influence membrane fluidity and lipid raft organization, thereby modulating receptor-mediated signaling [7,93].

EPA and DHA are enzymatically converted through cyclooxygenase (COX), lipoxygenase (LOX), and cytochrome P450 pathways into a distinct class of bioactive lipid mediators known as specialized pro-resolving mediators (SPMs). This group includes resolvins (E- and D-series), protectins, and maresins, which orchestrate the active resolution phase of inflammation by suppressing nuclear factor-κB (NF-κB) signaling, reducing neutrophil infiltration, enhancing macrophage-mediated efferocytosis, and promoting the clearance of apoptotic cells [93,94].

These SPMs exert their effects via specific G-protein–coupled receptors such as chemerin receptor 23 (ChemR23), G-protein-coupled receptor 32 (GPR32), and lipoxin A4 receptor / formyl peptide receptor 2 (ALX/FPR2), thereby initiating transcriptional programs that restore tissue homeostasis, limit collateral tissue damage, and prevent chronic inflammatory responses [95].

In this context, the role of omega-3 fatty acids becomes particularly relevant during aging, a state associated with a persistent low-grade inflammatory phenotype known as inflammaging. This process is characterized by elevated systemic levels of pro-inflammatory cytokines such as IL-6, TNF-α, and C-reactive protein (CRP), coupled with impaired resolution mechanisms [96].

Experimental and clinical studies have demonstrated that EPA and DHA supplementation in older adults can restore resolution capacity by elevating the circulating levels of pro-resolving lipid mediators. Such interventions are linked to enhanced macrophage phagocytic activity, improved T cell responsiveness, reduced expression of senescence-associated markers, and support of essential cellular processes, including autophagy and mitochondrial maintenance, both of which are vital for sustaining immune function in the aging host [97,98].

Beyond immunosenescence, omega-3 PUFAs have demonstrated potential clinical benefits in infectious diseases, particularly in reducing the incidence and severity of upper respiratory tract infections (URTIs) [99]. Μeta-analysis of randomized controlled trials (RCTs) concluded that the supplementation of 1.71 ± 0.89 g (ALA) and 0.11 ± 0.21 g (EPA + DHA) decreases the risk of respiratory infections, possibly through modulation of mucosal immunity and improved T helper cell balance [100]. During the COVID-19 pandemic, observational data suggested an inverse correlation between omega-3 index levels and circulating pro-inflammatory cytokines such as IL-1β, IL-6, and IL-8. Furthermore, higher omega-3 status was associated with improved clinical outcomes, including reduced hospitalization rates and accelerated recovery time, underscoring the potential role of these fatty acids in modulating host responses to viral infections [101].

Despite their established biological activity, the integration of omega-3 fatty acids into functional food products presents challenges, primarily due to their chemical instability and limited bioavailability. Recent advances in formulation technologies, such as nano mulsions, liposomes, and microencapsulation, have significantly improved the oxidative stability, palatability, and gastrointestinal absorption of EPA and DHA in various food matrices, including dairy-based products and nutritional supplements [102].

### 3.5. Food-Derived Bioactive Peptides: Molecular Mechanisms and Immunomodulatory Potential

Food-derived bioactive peptides generated from dietary proteins have emerged as important regulators of the human immune system, acting through highly specific molecular pathways. In recent years, research has focused on identifying distinct peptides—such as lactoferricin, β-casomorphin-7, and ovotransferrin-derived fragments—as well as amino acids like glutamine and arginine, all of which modulate immune cell activity and inflammatory processes [103]. These peptides, produced via enzymatic hydrolysis or fermentation, are abundant in milk, eggs, fish, and plant proteins, and exhibit unique structural motifs that enable receptor-mediated signaling in immune tissues. Lactoferricin, a peptide derived from the N-terminal region of bovine lactoferrin, has demonstrated robust immunoregulatory actions by suppressing pro-inflammatory cytokines (e.g., TNF-α, IL-6) and upregulating anti-inflammatory mediators, such as IL-10, via interaction with Toll-like receptor 4 (TLR4) on macrophages. In vitro studies confirm that lactoferricin can also inhibit the nuclear translocation of NF-κB, reducing the transcription of key inflammatory genes [104]. Similarly, β-casomorphin-7, released during gastrointestinal digestion of bovine β-casein, has been shown to interact with μ-opioid receptors on immune cells, modulating lymphocyte proliferation and dampening Th1-mediated responses—a mechanism relevant in gut-associated immune homeostasis [105].

Fish-derived peptides, particularly those isolated from salmon and sardine proteins, are rich in amino acids such as lysine and arginine, which act as precursors for nitric oxide synthesis and promote macrophage phagocytic activity [106]. The peptide Leu-Ser-Gly-Tyr-Gly-Pro-Asn-Arg (LSGYGPNR), identified in sardine muscle hydrolysates, has been shown in recent animal models to increase splenic NK cell cytotoxicity and enhance interferon-γ (IFN-γ) production, indicating potential adjuvant properties for mucosal immune defense [107].

Egg-derived peptides, including ovotransferrin fragments and the pentapeptide IRW (Ile-Arg-Trp) from egg white, have recently attracted attention for their dual antioxidant and immunomodulatory actions [108]. In a 2023 murine colitis model, egg white peptides improved body weight and colonic integrity, attenuated the inflammatory response, restored gut microbiota balance, and normalized short-chain fatty acid (SCFA) levels [109].

Human studies, though limited, are promising. A randomized controlled trial in elderly subjects supplemented with β-lactolin, a whey-derived Gly-Thr-Trp-Tyr lactopeptide, showed activation of the dopaminergic system, improvement in cognitive performance, and prevention of Alzheimer’s pathology in a rodent model [110]. Similarly, glutamine supplementation—alone or in peptide-rich hydrolysates—has been shown to enhance gut mucosal immunity and reduce infection rates in hospitalized patients by promoting intestinal intraepithelial lymphocyte differentiation and stimulating secretory IgA synthesis [111]. However, the translational efficacy of bioactive peptides is influenced by factors such as sequence specificity, gastrointestinal stability, and individual microbiome composition [9].

In summary, lactoferricin, β-casomorphin-7, fish-derived LSGYGPNR, and egg white IRW peptides represent potent immunomodulators with potential clinical applications in reducing inflammation and supporting host defense. Their multifaceted molecular actions—including cytokine regulation, immune cell activation, and maintenance of barrier integrity—underscore their importance in the development of next-generation functional foods and precision immunonutrition.

### 3.6. Probiotics and Gut–Immune Axis Modulation

Probiotics—live microorganisms which, when administered in sufficient quantities, confer health benefits to the host—modulate gut-associated lymphoid tissue (GALT), a key immunological niche housing the majority of the body’s immune cell population. By interacting with GALT, probiotics influence both mucosal and systemic immune responses [112]. Probiotic strains such as *Lactobacillus rhamnosus* GG, *Bifidobacterium lactis*, and *Saccharomyces boulardii* enhance mucosal barrier integrity by stimulating the production of tight junction proteins and mucins, preventing translocation of pathogens and endotoxins [113].

At the immunological level, probiotics modulate the balance between pro- and anti-inflammatory cytokines by inducing interleukin-10 (IL-10) and transforming growth factor-beta (TGF-β), which promote regulatory T cell differentiation and suppress excessive inflammatory responses [114]. They also activate dendritic cells, enhancing antigen presentation and promoting Th1 responses critical for antiviral immunity [115]. Probiotics increase the secretion of secretory immunoglobulin A (sIgA), which neutralizes pathogens at mucosal surfaces and contributes to immune exclusion [116].

Multiple randomized controlled trials (RCTs) report that probiotic supplementation reduces the incidence, duration, and severity of upper respiratory tract infections (URTIs) in children and adults by enhancing interferon production and modulating toll-like receptor (TLR) signaling pathways [117]. Furthermore, probiotics may enhance vaccine efficacy by promoting antigen-specific IgG and IgA responses, potentially serving as natural adjuvants [118].

**Figure 2:**
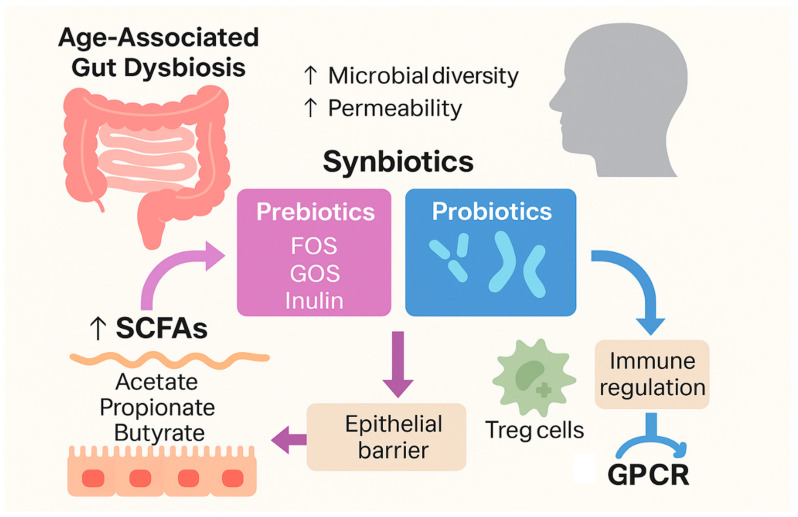
Mechanisms of action of probiotics/prebiotics/synbiotics in the immune system. ↑: increases/stimulates.

Age-associated gut dysbiosis—characterized by decreased microbial diversity and increased permeability—contributes to systemic endotoxemia and chronic immune activation. Prebiotics are non-digestible food ingredients—primarily oligosaccharides such as fructooligosaccharides (FOS), galactooligosaccharides (GOS), and inulin—that 118–120 combinations of probiotics and prebiotics—offer synergistic benefits by enhancing probiotic survival and colonization while simultaneously providing fermentable substrates for sustained metabolic activity. Promoting the proliferation of probiotic bacteria such as Bifidobacterium and Lactobacillus species leads to the production of short-chain fatty acids (SCFAs) like acetate, propionate, and butyrate. SCFAs serve as energy substrates for colonocytes, enhance epithelial barrier integrity, and exert systemic immunomodulatory effects through G protein-coupled receptor (GPCR) signaling (Figure 2). Butyrate has been shown to induce Treg cell differentiation, suppress pro-inflammatory Th17 cell responses, and inhibit histone deacetylases, thereby influencing gene expression involved in immune regulation [119,120,121].

Meta-analyses and RCTs in elderly cohorts demonstrate that symbiotic interventions improve vaccine efficacy, reduce respiratory infection rates, and modulate inflammatory biomarkers, thus enhancing immunological resilience [122], (Table 1).

**Table 1:** Functional food bioactives and their immunomodulatory properties.

Bioactive Compound	Dietary Source	Immune Effects	Evidence Type	Reference
Vitamin D	Fortified dairy, fish oil	↑ Cathelicidin, ↑ Tregs, ↓ IL-6, TNF-α, IFN-γ	Clinical trial, review	[41,42,43,44,45]
Vitamin C	Citrus fruits, kiwifruit, bell peppers	↓ ROS, ↑ phagocytosis, ↑ lymphocyte proliferation, ↓ NF-κB activity	Clinical studies, mechanistic	[46,47,48,49]
Vitamin E	Nuts, seeds, plant oils, spinach	↑ T cell response, ↓ oxidative damage	RCTs in elderly, mechanistic	[50,51,52]
Zinc	Shellfish, red meat, legumes	↑ T cell maturation, ↓ oxidative stress, ↑ NK and macrophage activity	RCTs, aging populations	[53,54,55,56,57,58]
Selenium	Brazil nuts, seafood	↑ NK cell function, ↑ antioxidant selenoproteins, ↓ ROS	Mechanistic studies, reviews	[59,60,61,62]
Iron	Red meat, liver, lentils, fortified cereals	Essential for T/B cell proliferation, ↑ phagocytosis, modulates redox balance	Clinical trials, supplementation studies	[63,64,65]
Polyphenols	Quercetin-Apples, onions, berries EGCG-Green tea Resveratrol Red grapes, wine	↓ Mast cell activation, ↑ antiviral interferon response ↑ Treg differentiation, ↓ NF-κB pathway ↑ SIRT1, ↓ age-associated inflammation	In vitro, animal model Review, in vivo Animal model	[66,67,68,69,70,71,72,73,74,75,76,77,78]
Curcumin	Turmeric (Curcuma longa)	↓ COX-2, ↓ iNOS, modulation of JAK/STAT and NLRP3 inflammasome, ↓ pro-inflammatory mediators, ↑ macrophage phagocytosis and antigen presentation, ↑ NK cytotoxicity, antioxidant properties	In vitro, in vivo, clinical potential	[79,80,81,82,83]
Flavonoids (Quercetin, Hesperidin)	Fruits, onions, apples, citrus	↓ viral entry/replication, ↓ mast cell activation and histamine release, ↓ MAPK and NF-κB signaling, ↓ cytokine storm. Hesperidin: ↑ endothelial function, ↑Nitric oxide (NO) synthesis, ↓ ICAM-1/VCAM-1, ↓ vascular inflammation	In vitro, animal models, mechanistic studies	[11,84,86]
Carotenoids (β-carotene, lycopene, lutein)	Carrots, tomatoes, leafy greens	↑ Retinoic acid signaling, ↓ IL-8, ↓ CRP, ↑ lymphocyte activity	Observational studies, mechanistic data	[87,88,89,90,91,92]
Omega-3 (EPA/DHA)	Fatty fish, algae oil	↑ Specialized pro-resolving mediators (SPMs), ↓ IL-6, ↑ macrophage efferocytosis	Meta-analysis, RCT	[93,94,95,96,97,98,99,100,101]
Peptides	Milk, fish, eggs, nuts, legumes	↓ TNF-α, ↓ IL-6, ↑ IL-10 Modulation of Th1/Th2 balance, ↓ pro-inflammatory cytokines ↑ NK cytotoxicity, ↑ IFN-γ ↓ IL-1β, ↑ Treg, improved gut barrier ↑ CD4+ T cells, ↑ Gut mucosal immunity, ↓ infections ↑ Macrophage activity, ↑ phagocytosis	Review, in vivo	[9,104,105,106,107,108,109,110,111,112]
Probiotics (L. *plantarum*)	Fermented dairy, kefir	↑ Mucosal IgA, ↑ IL-10, ↑ TGF-β, ↓ LPS-induced inflammation	Clinical trial, meta-analysis	[112,113,114,115,116,117,118,119,120,121,122]
Prebiotics (inulin, FOS)	Chicory root, onions	↑ SCFA (butyrate), ↑ gut Treg activity	Human intervention	

↑: increases/stimulates; ↓: decreases/inhibits.

Overall, incorporating well-characterized probiotic strains, targeted prebiotics, and rationally designed synbiotic formulations into functional foods represents a promising strategy for enhancing immune resilience and reducing the burden of infectious and inflammatory diseases across diverse populations.

### 3.7. Synergistic Interactions and Challenges in Functional Food Development

Synergistic interactions between functional food ingredients may provide amplified immunomodulatory effects. A well-established example is the ability of vitamin C to enhance non-heme iron absorption, thereby improving both hematological status and immune responses [123,124,125]. Probiotics and polyphenols also exhibit reciprocal interactions: probiotics metabolize polyphenols into bioactive metabolites, while polyphenols selectively stimulate beneficial microbial taxa [126,127]. Moreover, combined supplementation with omega-3 fatty acids and vitamin D has been associated with reduced systemic inflammation and improved immune markers in patients with chronic diseases [128]. These findings suggest that combinatorial dietary strategies may yield greater benefits than single compounds alone [129].

Functional foods enriched with immune-supportive ingredients offer a sustainable, translational approach to improving health, especially in aging societies challenged by infectious threats and inflammatory chronic diseases. Evidence from large cohorts (e.g., Mediterranean diet interventions) shows that dietary patterns rich in micronutrients and polyphenols correlate with reduced inflammaging markers and improved immune competence [130].

Novel delivery systems—such as nanoencapsulation and advanced emulsions—can enhance bioavailability of labile compounds like polyphenols and EPA/DHA, expanding the scope of food-based immunonutrition [131,132]. Moreover, interventions targeting cellular senescence through compounds like resveratrol may support both immune function and longevity pathways [133].

Functional foods enriched with these micronutrients are increasingly investigated for their potential to modulate immune responses, reduce inflammation, and protect against infectious diseases [134]. Clinical trials with fortified dairy products, cereals, and beverages have demonstrated improvements in biomarkers of immune function and reduced incidence of common infections [135,136].

Furthermore, polyphenols can beneficially modulate gut microbiota composition by promoting the growth of probiotic strains such as Lactobacillus and Bifidobacterium, while probiotics can enhance the bioavailability and metabolism of polyphenols into active phenolic metabolites—a phenomenon described as the “polyphenol–probiotic axis” [126]. This bidirectional relationship contributes to mucosal immune homeostasis, improved barrier function, and reduced systemic inflammation.

Similarly, ω3 fatty acids, carotenoids, and polyphenols co-regulate oxidative stress pathways by modulating redox-sensitive transcription factors such as NF-κB and Nrf2. Additionally, the co-formulation with antioxidant compounds such as vitamin E or polyphenols has shown synergistic effects, enhancing both the bioefficacy and the anti-inflammatory potential of omega-3–enriched products. These developments support the broader use of LC-PUFAs as functional immunonutrients with relevance in aging, infection control, and chronic disease prevention [137].

This complementary action attenuates pro-inf8ammatory signaling and enhances cellular resilience against oxidative insults, which are critical in aging populations and chronic inflammatory conditions [138]. The concurrent presence of these bioactive components in multicomponent functional foods offers a broader spectrum of action and more robust immunological outcomes than single-agent formulations.

Aging-associated immune dysregulation—including thymic involution, reduced naïve T cell populations, and chronic activation of inflammatory pathways—can be counteracted by micronutrients. Vitamins D and E, zinc, and selenium exert coordinated effects on immune cell function and redox homeostasis: Vitamin D enhances antimicrobial peptide production and regulatory T-cell activation; zinc supports T-cell thymic maturation and NK cell cytotoxicity; selenium is essential for antioxidant selenoprotein activity [139,140]. Concurrently, omega-3 fatty acids (EPA and DHA) foster resolution of inflammation via biosynthesis of specialized pro-resolving lipid mediators (SPMs), enhancing efferocytosis, and downregulating NF-κB–driven cytokine expression [141].

This micronutrient-lipid synergy is particularly relevant in older populations, where dietary deficiencies are common due to altered absorption, polypharmacy, and comorbidities. Optimizing these nutrient levels through diet or supplementation has shown beneficial effects on immune markers, inflammation resolution, and vaccine responses in the elderly [142,143]. Furthermore, clinical trial evidence indicates that functional food ingredients exert population-specific effects. For example, probiotic supplementation has been shown to reduce respiratory tract infection incidence in children [144,145], while vitamin D improves vaccine responses in elderly individuals [146]. In patients with chronic diseases such as cardiovascular and metabolic disorders, omega-3 fatty acids attenuate inflammation and improve immune function [147]. These examples demonstrate that population context is a critical determinant of translational relevance.

Although numerous studies support the beneficial effects of functional food ingredients on immune responses, the evidence is not uniformly consistent. For instance, vitamin D supplementation has been associated with reduced risk of respiratory infections in meta-analyses [45], yet some large, randomized trials failed to confirm significant effects [148], suggesting that outcomes may depend on baseline status, dosage, and regimen. Similarly, while certain probiotics have been shown to enhance vaccine responses, systematic reviews highlight heterogeneous findings and low overall certainty of evidence [149]. Curcumin represents another example: although preclinical and pilot studies demonstrate promising immunomodulatory and anti-inflammatory properties, especially in metabolic and oncological settings [82,83], robust large-scale RCTs remain lacking. These contradictions emphasize the importance of cautious interpretation and highlight the need for standardized, high-quality clinical trials to strengthen the evidence base.

Despite their promising benefits, several translational challenges limit the widespread application of bioactive-rich functional foods. Low bioavailability due to poor solubility, rapid metabolism, or degradation in the gastrointestinal tract remains a major barrier [98]. For instance, polyphenols like curcumin and EGCG have limited systemic absorption, necessitating the development of novel delivery systems such as nanoemulsions, liposomal encapsulation, or complexation with cyclodextrins to enhance stability and tissue targeting [150]. Translational applications of functional food ingredients raise important practical questions regarding the mode and duration of intake. Evidence from clinical trials suggests that regular and sustained administration, rather than short-term or sporadic intake, is required to achieve significant immunomodulatory effects. For example, daily vitamin D supplementation over several months has been associated with reduced risk of respiratory infections [45,148], while long-term omega-3 fatty acid intake improves inflammatory markers and respiratory outcomes [94,98,100]. Similarly, probiotic supplementation for at least 8–12 weeks has been shown to reduce respiratory tract infection incidence in children and adults [117,122]. These findings underscore that anti-inflammatory bioactive compounds are most effective when consumed consistently, either through diet or supplementation, and that duration of intake is a key determinant of outcomes. However, heterogeneity in study protocols highlights the need for further well-designed, long-term clinical trials to establish optimal regimens.

Another critical factor is the high interindividual variability in response to bioactive compounds, influenced by host genetics, microbiome composition, dietary patterns, and disease status [151]. Such variability underscores the need for precision nutrition approaches that integrate omics technologies (genomics, metabolomics, microbiomics) to tailor functional food interventions based on individual immune phenotypes.

Furthermore, regulatory and labeling inconsistencies across regions challenge the standardization and communication of health claims related to immunomodulatory foods. There is a pressing need for well-powered, long-term randomized clinical trials with immunological endpoints to substantiate efficacy and safety [95]. Ethical and scientific rigor in study design, alongside systems biology modeling, will be essential to elucidate dose–response relationships, bioactive–microbiome–immune interactions, and mechanistic pathways.

### 3.8. Heterogeneity of Clinical Evidence: Challenges and Opportunities

Despite mechanistic plausibility, clinical outcomes across functional food trials remain inconsistent. Heterogeneity arises from variability in dosage, bioavailability, participant baseline nutritional status, and genetic factors (e.g., polymorphisms affecting nutrient metabolism) [152]. Additionally, single-nutrient interventions often fail to account for synergistic interactions or antagonisms between compounds, which can significantly alter therapeutic efficacy [129].

Addressing these limitations requires multi-modal study designs, combining multi-nutrient interventions with systems biology approaches—such as nutrigenomics, metabolomics, and microbiome sequencing—to personalize immunonutrition strategies based on individual biomarker profiles [153].

Beyond the intrinsic bioactivity of nutraceuticals, recent research has increasingly focused on innovative delivery platforms designed to improve their stability, bioavailability, and targeted efficacy. Strategies such as liposomal encapsulation, nano- and micro-emulsions, and protein- or polymer-based carriers have been explored to optimize absorption and ensure controlled release of functional food ingredients [36,102,131,132,150,151]. While a detailed tabular summary of these systems would extend beyond the primary scope of this review, acknowledging these advances highlights their potential to further enhance the immunomodulatory benefits of nutraceuticals and supports the growing relevance of formulation science in immunonutrition.

## 4. Materials and Methods

This review was conducted in accordance with the Preferred Reporting Items for Systematic Reviews and Meta-Analyses (PRISMA) 2020 guidelines [154], in order to ensure transparency and reproducibility in the literature selection process. Although no protocol was preregistered, the study selection process was carried out systematically.

### 4.1. Information Sources and Search Strategy

A structured literature search was performed in major databases, including PubMed, Scopus, Web of Science, Cochrane Library, and ClinicalTrials.gov. Additional searches were conducted in the WHO ICTRP and other registries. Search covered the period between January 2000 and February 2025. Keywords and Boolean operators included combinations of “functional foods”, “immune function”, “micronutrients”, “polyphenols”, “inflammaging”, “immunosenescence”, “gut–immune axis”, and “dietary interventions”.

### 4.2. Eligibility Criteria

We considered original articles, systematic reviews, and meta-analyses published in English that investigated the role of bioactive dietary components in immune modulation. Both preclinical (in vitro and in vivo animal) and clinical (human observational and interventional) studies were included. Exclusion criteria were studies unrelated to immune health, studies not involving functional foods or bioactive compounds, papers of poor methodological quality, inaccessible full-texts, and editorials or non-English papers.

### 4.3. Study Selection

All records were imported into a reference management system, and duplicates were removed. Two authors independently screened titles and abstracts, followed by full-text assessment of potentially eligible articles. Disagreements were resolved through discussion. After removal of 196 duplicates, 1050 articles were screened, and 170 were assessed in full text. Of these, 100 were excluded for reasons described in Supplementary Figure S1. Finally, 70 studies were included in the qualitative synthesis.

### 4.4. Data Extraction and Synthesis

Data were extracted and categorized into thematic domains: micronutrients, polyphenols, gut microbiota modulators (probiotics and prebiotics), peptides, carotenoids, omega-3 fatty acids, and aging-related immune responses. Extracted variables included compound type, source, immunomodulatory mechanism, study type, and outcomes. Results were synthesized narratively and organized by compound class and mechanistic pathways.

### 4.5. Risk of Bias and Certainty of Evidence

As this is a narrative review with systematic search elements, no formal risk-of-bias assessment (e.g., Cochrane RoB2, ROBINS-I) or certainty grading (e.g., GRADE) was performed. Instead, emphasis was placed on transparency in reporting the search, selection, and synthesis processes. Where relevant, the strengths and limitations of evidence were qualitatively appraised in the Discussion.

### 4.6. PRISMA Documentation

The study selection process is presented in the PRISMA 2020 flow diagram (Supplementary Figure S1). An adapted PRISMA 2020 checklist for narrative reviews is provided in Supplementary Table S1, clarifying which items are not applicable to this review type.

## 5. Conclusions

The evidence synthesized in this review underscores the significant role of functional food ingredients in maintaining and enhancing immune competence, particularly in the context of aging and chronic low-grade inflammation. Micronutrients such as vitamins C, D, and E, as well as zinc and selenium, along with bioactive compounds like polyphenols, omega-3 fatty acids, and modulators of the gut microbiota, demonstrate measurable benefits in modulating both innate and adaptive immune responses. Their immunoprotective effects are attributed to a range of mechanisms, including antioxidant defense, regulation of inflammatory signaling pathways (e.g., NF-κB, NLRP3 inflammasome), support of epithelial barrier function, and enhancement of T and B cell responses. These effects are particularly relevant in older adults, who exhibit greater vulnerability to infections and immunosenescence. Despite promising findings, heterogeneity across clinical studies underscores the need for standardized, longitudinal, and personalized interventions, considering individual variability in nutrient status, genetic factors, and microbiome composition. Integration of multi-omics tools, real-time biomarker monitoring, and food delivery technologies (e.g., nanoencapsulation) may enhance the translational potential of functional foods in preventive health.

In conclusion, functional foods hold promise as adjunct strategies to support immune health. However, their safety, cost-effectiveness, and sustainability cannot yet be assumed and should be carefully evaluated in real-world settings. Differences in accessibility, regulatory frameworks, and population-specific responses must also be taken into account. Future research, including large-scale randomized trials and health-economic analyses, is required to confirm their feasibility and to guide evidence-based recommendations.

## Supplementary Materials

The following supporting information can be downloaded at: https://www.mdpi.com/article/10.3390/ijms26178408/s1.

## Abbreviations

The following abbreviations are used in this manuscript:

NF-κB	Nuclear Factor-kappa B
MAPK	Mitogen-Activated Protein Kinases
Nrf2	Nuclear Factor Erythroid 2–Related Factor 2
FOS	Fructooligosaccharides
SCFA	Short-Chain Fatty Acids
Treg	Regulatory T Cells
EPA	Eicosapentaenoic Acid
DHA	Docosahexaenoic Acid
SPMs	Specialized Pro-Resolving Mediators
VDR	Vitamin D Receptor
IL	Interleukin
TNF-α	Tumor Necrosis Factor-Alpha
ROS	Reactive Oxygen Species
NK	Natural Killer (Cells)
HO-1	Like Heme Oxygenase-1
GPx	Glutathione Peroxidase
EGCG	Epigallocatechin-3-Gallate
AMPK	AMP-Activated Protein Kinase
SIRT1	Sirtuin 1
mTOR	Mechanistic Target of Rapamycin
SASP	Senescence-Associated Secretory Phenotype
COX-2	Cyclooxygenase-2
iNOS	Inducible Nitric Oxide Synthase
JAK/STAT	Janus Kinase / Signal Transducer and Activator of Transcription
NLRP3	NOD-, LRR- and Pyrin Domain-Containing Protein 3
ICAM-1	Intercellular Adhesion Molecule 1
VCAM-1	Vascular Cell Adhesion Molecule 1
RARs	Retinoic Acid Receptors
MCP-1	Monocyte Chemoattractant Protein-1
LC-PUFAs	Long-Chain Polyunsaturated Fatty Acids
LOX	Lipoxygenase
ChemR23	Chemerin Receptor 23
GPR32	G-Protein-Coupled Receptor 32
ALX/FPR2	Lipoxin A4 Receptor / Formyl Peptide Receptor 2
CRP	C-Reactive Protein
URTIs	Upper Respiratory Tract Infections
RCTs	Randomized Controlled Trials
GALT	Gut-Associated Lymphoid Tissue
TGF-β	Transforming Growth Factor-Beta
Th1	T Helper Type 1 (Cells)
sIgA	Secretory Immunoglobulin A
IFN-γ	Interferon Gamma
