# Correction: Skenderidou et al. Functional Food Ingredients Enhancing Immune Health: A Systematic Review. *Int. J. Mol. Sci.* 2025, *26*, 8408

**DOI:** 10.3390/ijms262311636

**Published:** 2025-12-01

**Authors:** Irene Skenderidou, Stefanos Leontopoulos, Prodromos Skenderidis

**Affiliations:** 1 School of MΕd, University of Nicosia, 46 Makedonitissas Avenue, CY-2417 Nicosia, Cyprus; skenderidou.e@live.unic.ac.cy; 2 School of Applied Arts and Sustainable Design, Hellenic Open University, Parodos Aristotelous 18, 26335 Patras, Greece; sleontopoulos@uth.gr; 3 School of Sciences & Technology (SST), Hellenic Open University, Parodos Aristotelous 18, 26335 Patras, Greece

In the original publication [1], the article type was incorrectly classified. The review should be Systematic Review. According to the requirements of the Systematic Review, the authors made the following corrections:

## Title

The title has been changed to “Functional Food Ingredients Enhancing Immune Health: A Systematic Review”.

## Figure Correction

The change in article type to Systematic Review caused a change in the position of Figure S1. Move Figure S1. PRISMA flow diagram to the body text section and change the title to Figure 1. PRISMA flow diagram. The only change is the addition of a figure that was in the Supplementary Materials on the materials and methods section.

**Figure 1:**
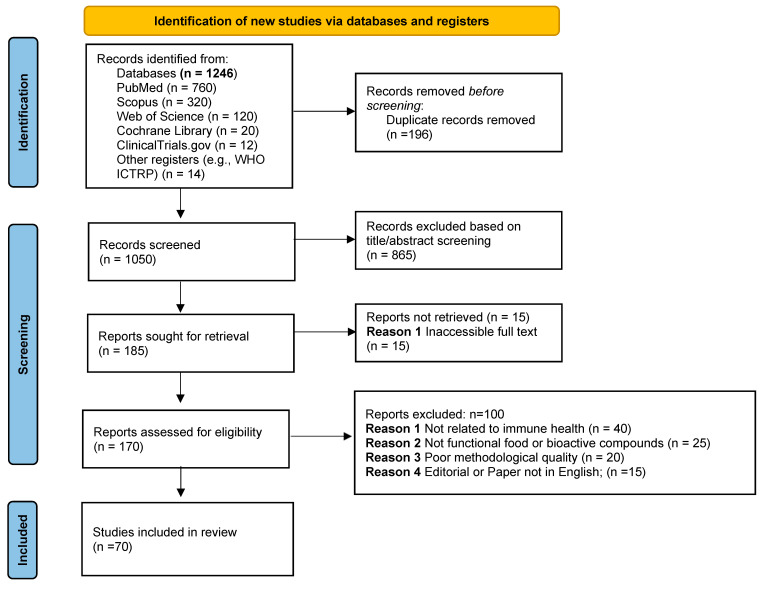
PRISMA flow diagram.

## Error in Table

The change in article type to Systematic Review caused Table S1. PRISMA 2020 Checklist (Adapted for Narrative Review) change to Table S1. PRISMA 2020 Checklist. The authors state that the scientific conclusions are unaffected.

## Text Correction

The structure of the article has been rearranged in accordance with journal requirements 1. Introduction, 2. Methods, 3. Results and Discussion, 4. Conclusions.

The authors state that the scientific conclusions are unaffected. This correction was approved by the Academic Editor. The original publication has also been updated.
