# Supplementary material for: Functional Food Ingredients Enhancing Immune Health
Source: Int J Mol Sci. 2025 Aug 29;26(17):8408. doi: 10.3390/ijms26178408 (PMC12428461; doi:10.3390/ijms26178408)
Supplement: Supplementary file 1 [file ijms-26-08408-s001.zip › Table S1 PRISMA Checklist.pdf]

Table S1. PRISMA 2020 Checklist (Adapted for Narrative Review)

Section	Checklist Item	Status / Notes
TITLE	Identify the report as a systematic review.	✓ Title clearly mentions 'Review' and context of immune nutrition. Functional Food Ingredients Enhancing Immune Health – explicitly is a narrative with systematic search elements.
ABSTRACT	Structured summary including background, objectives, data sources, eligibility criteria, etc.	✓ Structured abstract summarizes background, methods, results, and conclusions.
INTRODUCTION - Rationale	Describe the rationale for the review in the context of existing knowledge.	✓ The introduction outlines the importance of functional foods in immune modulation.
INTRODUCTION - Objectives	Provide an explicit statement of the objective(s).	✓ Objective stated: evaluate the role of functional foods on immune health.
METHODS - Eligibility criteria	Specify inclusion and exclusion criteria.	✓ Included in vivo, in vitro, and clinical studies, systematic reviews, and meta-analyses between 2000–2025. English language only.
METHODS - Information sources	Specify all databases and sources searched.	✓ Databases: PubMed, Scopus, Web of Science, Cochrane Library, ClinicalTrials.gov, WHO ICTRP
METHODS - Search strategy	Present full search strategy for at least one database.	✗ Keywords included: 'functional foods', 'immune function', 'micronutrients', 'polyphenols', etc..
METHODS - Selection process	Describe methods used to select studies.	✓ Based on title, abstract, and full-text screening by authors. Manual filtering applied.
METHODS - Data collection process	Describe method of data extraction.	✓ Studies were categorized by thematic domains: micronutrients, polyphenols, gut microbiota modulators, and ageing-related immune responses.
METHODS - Data items	List and define all variables for which data were sought.	✓ Immunomodulatory outcomes, compound mechanisms, study type, and efficacy.

METHODS - Study risk of bias assessment	Specify methods used to assess risk of bias.	Not applicable; no formal risk-of-bias was conducted as this is a narrative review.
METHODS - Effect measures	State principal summary measures.	Not applicable-narrative synthesis-, no meta-analysis performed.
METHODS - Synthesis methods	Describe methods for combining results.	✓ Narrative synthesis of findings categorized by compound class and function.
METHODS - Reporting bias assessment	Assess risk of bias due to missing results.	Not assessed – narrative review design.
METHODS - Certainty assessment	Assess certainty of evidence.	Not applicable – narrative review without GRADE assessment.
RESULTS - Study selection	Give numbers of studies screened and included, with flow diagram.	✓ 1246 records identified, 196 duplicates removed, 1050 screened, 170 full-text assessed, 100 excluded. 70 studies included. Flow diagram provided (Figure S1).
RESULTS - Study characteristics	Present characteristics of included studies.	✓ Summarized in text and Table 1.
RESULTS - Risk of bias in studies	Present assessments of risk of bias.	Not applicable – narrative review.
RESULTS - Results of individual studies	Present results for all outcomes.	✓ Thematic findings presented.
DISCUSSION - Summary of evidence	Summarize main findings.	✓ Comprehensive synthesis of literature.
DISCUSSION - Limitations	Discuss limitations of included evidence.	✓ Limitations of heterogeneity and methodological variability discussed.
DISCUSSION - Conclusions	Provide interpretation of results.	✓ Detailed conclusion and future directions.
OTHER - Registration and protocol	Provide registration information.	Not registered – narrative review, protocol not applicable.
OTHER - Support	Describe sources of funding.	✓ Declared: No external funding.
OTHER - Competing interests	Declare competing interests.	✓ Declared: No conflict of interest.
OTHER - Availability of data	Availability of data, code, etc.	✓ Data drawn from public sources, all references provided.

Note: This checklist was compiled based on the manuscript contents and inferred literature flow.

Final inclusion count (n=70) matches the references cited. The PRISMA flow diagram is included in the main manuscript. No protocol was registered for this review.
