# Supplementary figures and images for: Functional Food Ingredients Enhancing Immune Health
Source: Int J Mol Sci. 2025 Aug 29;26(17):8408. doi: 10.3390/ijms26178408 (PMC12428461; doi:10.3390/ijms26178408)

Figure S1. PRISMA flow diagram.

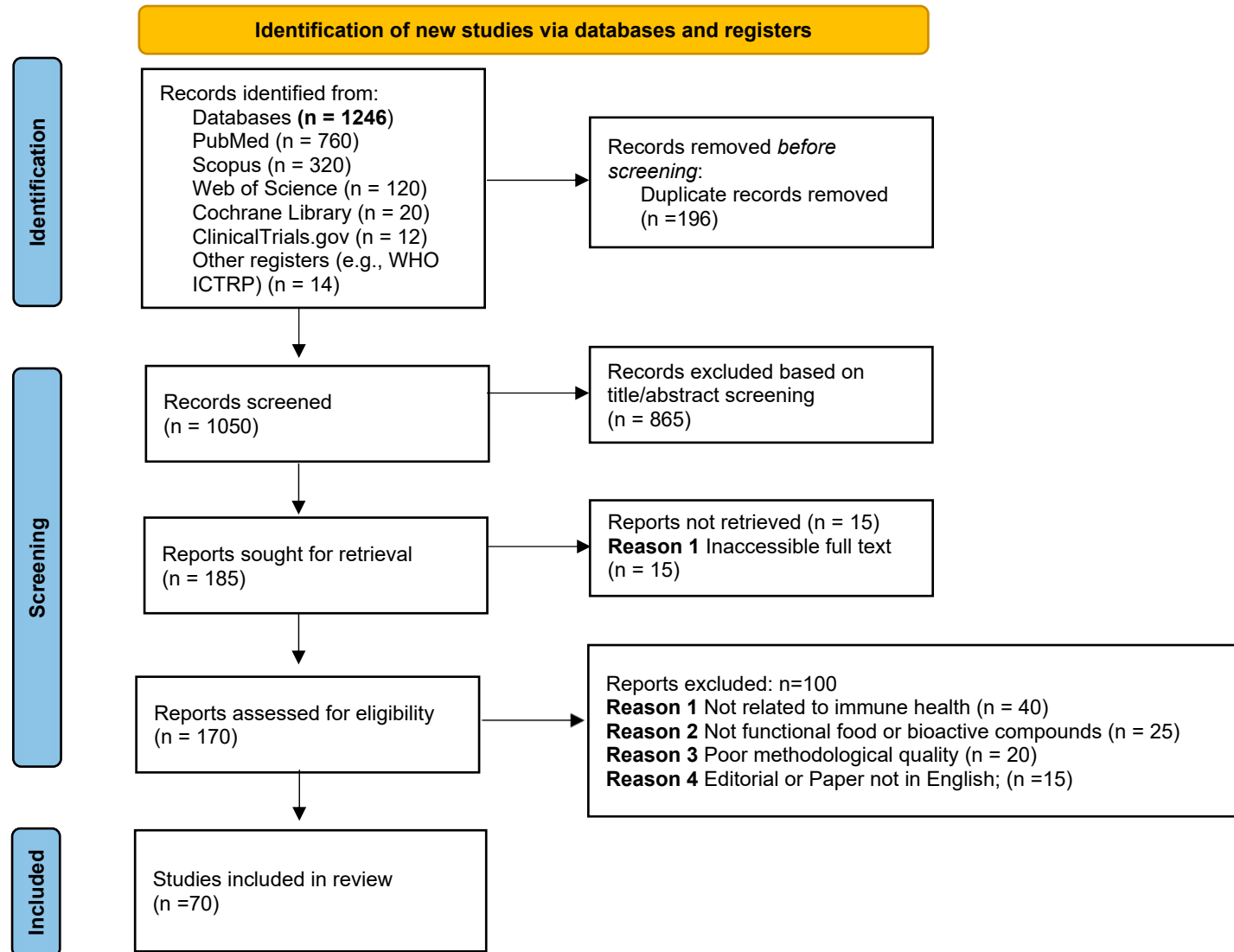

Supplement: Supplementary file 1 [file ijms-26-08408-s001.zip › Figure SF1 PRISMA 2020 flow_diagram.pdf]
